# Associations among height, body mass index and intelligence from age 11 to age 78 years

**DOI:** 10.1186/s12877-016-0340-0

**Published:** 2016-09-29

**Authors:** Mathew A. Harris, Caroline E. Brett, Ian J. Deary, John M. Starr

**Affiliations:** 1 Centre for Cognitive Ageing and Cognitive Epidemiology, University of Edinburgh, Edinburgh, UK; 2 Centre for Clinical Brain Sciences, University of Edinburgh, Edinburgh, UK; 3 School of Natural Sciences and Psychology, Liverpool John Moores University, Liverpool, UK; 4 Alzheimer Scotland Dementia Research Centre, University of Edinburgh, Edinburgh, UK

**Keywords:** Intelligence, Height, Body mass index, Ageing, Longitudinal study, 36-day sample

## Abstract

**Background:**

Intelligence is related to both height and body mass index (BMI) at various stages of life. Several studies have demonstrated longitudinal relationships between these measures, but none has established whether height and intelligence, or BMI and intelligence are linked from childhood through to older age.

**Methods:**

We assessed the relations between these measures over an interval of up to 67 years using data from the 36-Day Sample, an initially-representative sample of Scottish people born in 1936, assessed at age 11 years (*N* = 6,291) and again at 77–78 years (*N* = 722). This paper focuses on the 423 participants (6.7 % of the original sample) who provided relevant data in late adulthood.

**Results:**

Height and intelligence were significantly positively associated in childhood (*β* = .23) and late adulthood (*β* = .21–.29). Longitudinal correlations also showed that childhood intelligence predicted late-adulthood height (*β* = .20), and childhood height predicted late-adulthood cognitive ability (*β* = .12–.14). We observed no significant relationship between BMI and intelligence either in childhood or in late adulthood, nor any longitudinal association between the two in this sample.

**Conclusions:**

Our results on height and intelligence are the first to demonstrate that their relationship spans almost seven decades, from childhood through to late adulthood, and they call for further investigation into the mechanisms underlying this lifelong association.

**Electronic supplementary material:**

The online version of this article (doi:10.1186/s12877-016-0340-0) contains supplementary material, which is available to authorized users.

## Background

Height and intelligence, although seemingly very different measures, are empirically related [1, 2]. This relationship is present cross-sectionally in adults at their physical and intellectual peak, in childhood [3–5], and in late adulthood [6, 7]. Benyamin et al. [4] observed a correlation of .28 between height and intelligence phenotypes at age 11 years in Scottish children born in 1936, who participated in the Scottish Mental Survey 1947 (SMS1947). Some suggest that the relationship is largely genetic [8, 9], while others highlight the importance of childhood environmental factors in promoting both height and intelligence [10, 11]. Whichever is more important, height and intelligence seem to develop throughout childhood in parallel [5]. In late adulthood, declines in height and intelligence may again share common underlying mechanisms, although the specific genetic and environmental factors may be quite different to those promoting development in childhood. For example, while genes promoting growth and environmental factors such as nutrition may promote both physical and neurological development in childhood, genes associated with senescence and environmental factors such as stress may promote both physical and neurological decline. Furthermore, the relationship between height and intelligence in childhood [3] and in late adulthood [7] is not always the same for males and females, who show different patterns of development and decline. Therefore, while there is evidence of a relationship between height and intelligence at various stages of life, it is as yet unknown whether the two will be as closely related longitudinally.

Body mass index (BMI) and intelligence appear to be negatively correlated, particularly in late adulthood [12–14]. However, rather than being attributable to common underlying mechanisms and parallel development, this relationship seems to be serial, although the direction of causation is disputed. Some suggest that obesity leads to vascular risk factors that cause or exacerbate neurodegeneration, thereby resulting in greater cognitive decline [14–16]. Others argue that more intelligent people generally maintain a more normal BMI, as they are more likely to eat a healthy diet and to exercise regularly [17–19]. Evidence for both accounts has been derived from longitudinal studies, for example Halkjær et al. [17] studied men in early adulthood (mean age 19 years) and again approximately 24 years later, and found that lower intelligence at baseline predicted increases in BMI and the development of obesity. On the other hand, Dahl et al. [15] found that being overweight at around age 40 years predicted greater cognitive decline after age 60 years. However, no previous studies have investigated the relationship between BMI and intelligence from childhood right through to late adulthood. Additionally, not all prior studies suggest that BMI and intelligence are related; for example, Benyamin et al. [4] observed no significant phenotypic correlation in children of the SMS1947.

In the present study, we assessed the relationship between height and intelligence, and between BMI and intelligence, at age 11 years, at age 77–78 years, and over the 67-year interval in between. We made use of data from the 36-Day Sample, an initially representative sample of participants from the almost whole-year-of-birth Scottish Mental Survey 1947 [20–22], described in further detail below. We aimed firstly to test whether, in the 36-Day Sample, as in other samples, there are cross-sectional associations between height and intelligence, and between BMI and intelligence, in both childhood and late adulthood, using measures of both crystallised and fluid cognitive ability in late adulthood. We also set out to assess the longitudinal relations between intelligence and height and BMI over a much longer interval than previously studied, from childhood through to late adulthood. Due to the theorised common factors underlying height and intelligence differences, and to the apparent effects of intelligence on BMI and/or vice versa, we expected the relationships between each physical metric and intelligence to be observable even over a period as long as 67 years. We also explored the possibility that either of these longitudinal relationships was dependent upon or influenced by sex.

## Methods

### Participants

The second Scottish Mental Survey (SMS1947) took place on 4th June 1947 [23]. Almost all children born in 1936 and present at schools in Scotland on that day completed the Moray House Test No. 12 (MHT) [24], a validated test of intelligence. A representative sample of the same cohort (including those absent from school on the day of the SMS1947) were chosen to complete a short sociological schedule according to their date of birth being on one of the first three days of the month. Among other things, the sociological schedule recorded the heights and weights of the 6,291 members of the ‘36-Day Sample’. A smaller sub-sample, the ‘6-Day Sample’ (those born on the first day of February, April, June, August, October or December), were examined in much greater depth until 1963.

**Fig. 1:**
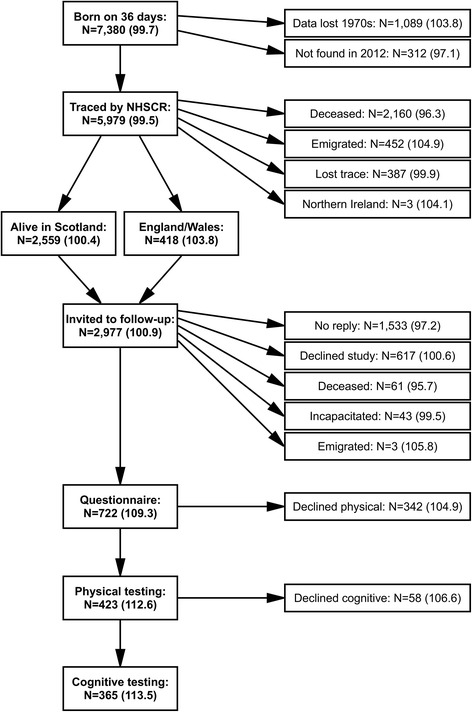
Participation in the 36-Day Sample follow-up study. NHSCR = UK National Health Service Central Register. Mean IQ (derived from Moray House Test score, standardised across all participants in the Scottish Mental Survey of 1947) is included in parentheses for each subset of participants. One member of the 36-Day Sample whose childhood data were lost in the 1970s and one other who was resident outside the UK volunteered to participate in the follow-up study after finding out about it from other members of the 36-Day Sample who had been invited to the follow-up

In 2012, as many members of the 6-Day Sample as possible were traced through the United Kingdom National Health Service Central Register (NHSCR). Of the original 1,208 sample members, 634 were found to be still alive and resident in Scotland, England or Wales; these individuals (as well as one other who had emigrated) were invited to participate in a follow-up study [25]. Starting later in 2012 and continuing through 2013, the 174 (92 female) who agreed to take part completed a detailed questionnaire booklet and physical testing [26], and 131 (72 female) also completed a subsequent telephone interview, incorporating a number of cognitive tests [22]. The remainder of the 36-Day Sample (*N* = 5,083) were traced through the NHSCR in 2013, and those alive and resident in Great Britain (*N* = 2,343) were invited to participate in the same follow-up study. An additional 548 (253 female) completed the questionnaire, 249 (99 female) completed physical testing in 2014 and 234 (96 female) had completed cognitive testing by mid-2015. The follow-up study selection process is illustrated in Fig. 1, showing that the two main reasons for the follow-up sample being so much smaller than the original 36-Day Sample were: i) a large proportion of the original sample had died before reaching their late seventies; ii) most of the original sample members who were traced and invited to the follow-up study simply did not agree to participate. Further details on members of the 36-Day Sample who participated in each stage of the study are also included in Table 1. Those who participated in the follow-up study were first fully informed about the study, and provided consent in writing. This paper focuses primarily on the 423 (191 female) participants (6.7 % of the original sample) who participated in the physical testing part of the follow-up study in late adulthood.

**Table 1:** Descriptive statistics of the 36-Day Sample in childhood and in older age

	36-Day Sample (1947)		Participated in SMS1947		Phys. tested (2012–14)		Cog. tested (2013–15)	
	M	SD	M	SD	M	SD	M	SD
*N*	6,291		5,742		423		365	
Sex (M/F)	3,112/3,179		2,849/2,893		232/191		199/166	
Age	11.0	0.3	11.0	0.3	77.7	0.9	78.2	0.9
Childhood								
Height (cm)	137.1	7.3	137.2	7.3	139.0	7.4	139.1	7.5
Weight (kg)	31.6	4.9	31.6	4.9	32.5	4.8	32.7	4.9
BMI	16.7	1.8	16.7	1.8	16.8	1.9	16.9	2.0
MHT IQ			100.0	15.0	111.5	10.8	112.5	10.5
Older-age								
Height (cm; men)					172.1	6.9	172.2	6.7
Height (cm; women)					157.9	6.1	157.9	5.8
Weight (kg)					75.1	14.6	74.9	14.3
BMI					27.2	4.4	27.2	4.3
NART (%)							68.1	16.4
RSPM (%)							54.5	12.9

*SMS1947* Scottish Mental Survey of 1947, *BMI* body mass index, *MHT IQ* Moray House Test intelligence quotient, *NART* National Adult Reading Test, *RSPM* Raven’s Standard Progressive Matrices. Sample summaries are provided for the entire 36-Day Sample in 1947, for those who participated in the SMS1947, for the follow-up participants who provided physical measurements in older age, and for those who also completed cognitive testing. There was a significant sex difference in late-adulthood height, which is therefore presented separately for men and women

### Measures

#### Height

Members of the 36-Day Sample had their heights measured as part of the sociological schedule, completed by their teachers shortly after the SMS1947 [27]. Surviving members of the 36-Day Sample who agreed to participate in the follow-up study measured their own height again in late adulthood, with a measuring tape and according to standard instructions we provided [25, 26]. We asked participants to remove any footwear or headgear, to stand up straight against a wall with their feet together, to place a flat box on top of their head, keeping it horizontal, and to mark the point on the wall at which the lower edge of the box touched it, then to measure the height of this mark using the provided tape. We also asked participants to seek assistance from a friend or relative in measuring their height in this way, which, in the majority of cases, they did. Childhood height was measured in inches, whereas late-adulthood height was recorded in the questionnaire booklet in whichever units participants preferred. All heights were converted into centimetres before analysis.

#### Weight

Members of the 36-Day Sample had their weights measured in childhood as part of the sociological schedule [27]. Follow-up study participants reported their weight in late adulthood, measured according to our instructions - first thing in the morning, wearing only light clothing. All recorded weights were converted into kilograms.

#### Body mass index

For each participant whose height and weight were both recorded, in childhood and/or in late adulthood, body mass index (BMI) was calculated by dividing weight in kilograms over squared height in metres [28].

#### Moray House Test

As above, almost all children born in 1936 and present at school on the day of the SMS1947 completed the Moray House Test No. 12 [23, 24]. The MHT is a group-administered test, has a time limit of 45 min, and has 71 numbered items. It assesses a range of cognitive abilities, with a preponderance of verbal reasoning items. The SMS1947 included 5,742 of the 36-Day Sample participants. For descriptive statistics, we derived an IQ-type score (MHT IQ) from each participant’s score on the MHT test by adjusting for age and then standardising scores, setting the mean to 100 and the standard deviation to 15.

#### National Adult Reading Test

During the telephone interview, participants completed the National Adult Reading Test (NART) [29] as a measure of late-adulthood verbal ability. The NART involves reading aloud a written list of 50 irregular words, producing a score corresponding to the number of words pronounced correctly. This reflects a more crystallised ability, being relatively well preserved in older age and even mild dementia [30]. The word list was sent to participants in advance by post, sealed in a second envelope until the time of the telephone interview. They were then instructed to open the envelope and read the list of words during the interview.

#### Raven’s Standard Progressive Matrices

Those who participated in the telephone interview also completed Raven’s Standard Progressive Matrices (RSPM) [31], a test of non-verbal reasoning. This test comprises five sets of twelve progressively more difficult non-verbal problems, each of which asks participants to select the missing piece of a two-dimensional pattern from six (sets A and B) or eight (sets C to E) possible options. The test booklet was also included in the sealed envelope sent to participants in advance of the telephone interview, and participants gave their responses verbally over the telephone to as many items as possible within 20 min. The number of correct responses was used as a measure of non-verbal reasoning in late adulthood. This was chosen as being a relatively fluid cognitive ability, tending to show greater decline into and throughout late adulthood, by contrast with the more crystallised ability assessed using the NART.

### Statistical analysis

Data were analysed in Matlab (R2013a; MathWorks, Natick, MA). As not all variables were normally distributed, we used generalised linear modelling to assess the bivariate associations between physical and cognitive measures in childhood, in late adulthood and over the 67-year interval. We first residualised all variables over age at measurement, capped outlying values at three times the standard deviation from the mean, and z-scored all variables in order to derive standardised *β* coefficients. We expected sex to affect the longitudinal relationship between height and intelligence due to large differences between men and women in height increase during adolescence, as well as in height decrease during late adulthood, but much less substantial sex differences in lifelong cognitive change. Some prior studies have also found sex specificity of a longitudinal relationship between height and intelligence [3, 7]. We therefore used unpaired t-tests to assess sex differences in height, intelligence and also BMI, and included sex and an interaction term in each of the generalised linear models. Where significant interactions were observed, those associations were also assessed separately for men and women. False discovery rate (FDR) [32] procedures were used to correct for multiple comparisons.

## Results

### Height and intelligence

Key descriptive statistics for participants included at each stage of the study – including their heights in childhood and, where available, in late adulthood – are presented in Table 1. The original 36-Day Sample was highly representative of the population, but those who participated in the follow-up study were significantly taller (*t*_6712_ = 5.18, *p* < .001) and heavier (*t*_6712_ = 3.66, *p* < .001) as children, with substantially higher IQs (*t*_6132_ = 14.92, p < .001). We used unpaired t-tests to test for sex differences in any of the height and intelligence variables, and found that men were significantly taller (*t*_416_ = 21.54, *p* < .001) and performed significantly better on the RSPM (*t*_357_ = 2.43, *p* = .02) in older age, while women had significantly higher IQs as children (*t*_393_ = 2.07, *p* = .04). After correcting for multiple comparisons, only the difference in older-age height remained significant.

In order to assess the representativeness of the sample included in the follow-up study, we assessed the relationship between height and IQ at age 11 in each of the sub-samples for which the data were available (Additional file 1: Figure S1, top). There was a modest significant positive correlation between childhood height and IQ in the original 36-Day Sample (*r* = .26, *p* < .001) and this correlation was still evident among those who provided physical measurements in older age (*r* = .23, *p* < .001) and those who completed cognitive testing (*r* = .23, *p* < .001). Although the strength of this correlation was slightly weaker in the two follow-up sub-samples, testing confirmed that these differences were not significant (all *p* > .527).

Focusing on those members of the 36-Day Sample involved in the follow-up study, we first assessed contemporaneous relations between height and intelligence (each residualised over age at measurement) in both childhood and late adulthood (Table 2). Controlling sex, age-11 height showed a significant relationship with MHT performance (*β* = .23, *p* < .001). This model also showed a significant effect of sex on MHT performance (*β* = .11, *p* = .02), but no significant interaction between childhood height and sex. In older age, there was a significant positive association between height and NART performance (*β* = .29, *p* < .001), with this model also showing a significant effect of sex (*β* = .24, *p* < .001), but no significant interaction effect. There was also a significant association between height and RSPM performance (*β* = .21, *p* = .01), but here no significant effect of sex, nor a significant interaction.

**Table 2:** Associations between height and intelligence in childhood, in older age, and from childhood to older age

Predictor	Outcome	*N*	Predictor		Sex		Interaction	
			*β*	*p*	*β*	*p*	*β*	*p*
Childhood height	Childhood MHT	375	**.23**	***	**.11**	**.02**	-.05	.32
Older-age height	Older-age NART	358	**.29**	**	**.24**	***	-.03	.58
	Older-age RSPM	356	**.21**	**.01**	.03	.73	.06	.25
Childhood height	Older-age height	396	**.47**	***	**-.73**	***	**-.06**	**.01**
Childhood MHT	Older-age NART	342	**.68**	***	-.05	.23	.02	.58
	Older-age RSPM	341	**.42**	***	**-.19**	***	.07	.17
Childhood height	Older-age NART	341	**.20**	***	.02	.72	-.01	.87
	Older-age RSPM	339	**.14**	**.01**	**-.15**	**	.04	.50
Childhood MHT	Older-age height	392	**.12**	**	**-.74**	***	.02	.62

*MHT* Moray House Test, *NART* National Adult Reading Test, *RSPM* Raven’s Standard Progressive Matrices. Statistics reported are standardised *β* coefficients and uncorrected p values derived from generalised linear models. All variables were residualised over age at measurement before modelling. Although variables are modelled as ‘predictor’ and ‘outcome’ variables, direction of causation is not implied, particularly for contemporaneous associations. Significant results at *p* < .05 are highlighted in bold; ** *p* < .01; *** *p* < .001

We next assessed the stability of both height and intelligence, and the association between them over the 66–67 years between the SMS1947 and the more recent follow-up study. As detailed in Table 2, there was a positive association between childhood height and older-age height (β = .47, p < .001). As expected, this model showed a large effect of sex (*β* = −.73, *p* < .001), as well as a significant interaction (*β* = −.06, *p* = .01). Although the interaction effect did not survive correction for multiple comparisons, we assessed the bivariate association separately for each sex group; childhood height showed a strong positive association with late adulthood height in both men (*β* = .71, *p* < .001) and women (*β* = .66, *p* < .001). There was no significant sex difference in the strength of this association. Furthermore, as observed previously in the 6-Day Sample [22], childhood intelligence significantly predicted both measures of late-adulthood cognitive ability (NART: *β* = .68, *p* < .001; RSPM: *β* = .42, *p* < .001). There was a significant effect of sex on RSPM performance (*β* = −.19, *p* < .001), but no significant interaction between childhood MHT performance and sex for either measure of older-age cognitive ability.

Assessing the longitudinal relationship between height and intelligence indicated that childhood height predicted better older-age NART performance (*β* = .20, *p* < .001; Table 2), with no significant effect of sex or interaction. Childhood height also predicted better older-age RSPM performance (*β* = .14, *p* = .01), but this model showed a similar effect of sex (*β* = −.15, *p* < .01), although no significant interaction. Childhood intelligence also predicted late adulthood height (*β* = .12, *p* < .01), but this model showed a larger effect of sex (*β* = −.74, *p* < .001), although no significant interaction. All significant associations and effects of sex remained significant after correcting for multiple comparisons.

### BMI and intelligence

Descriptive statistics representing the BMI of participants in each stage of the study are also included in Table 1, showing that all sub-samples were very similar. Unpaired t-tests revealed no significant sex differences in BMI either in childhood or in late adulthood (all *p* > .162). Nevertheless, Table 3 includes results for models incorporating sex as a covariate.

**Table 3:** Associations between body mass index and intelligence in childhood, in older age, and from childhood to older age

Predictor	Outcome	*N*	Predictor		Sex		Interaction	
			*β*	*p*	*β*	*p*	*β*	*p*
Childhood BMI	Childhood MHT	374	-.03	.58	**.12**	**.02**	-.01	.87
Older-age BMI	Older-age NART	356	-.10	.06	.03	.62	.03	.61
	Older-age RSPM	354	-.02	.65	**-.14**	**	-.07	.21
Childhood BMI	Older-age BMI	392	**.26**	***	.02	.73	.02	.67
Childhood BMI	Older-age NART	341	-.06	.26	.02	.76	-.02	.67
	Older-age RSPM	339	***	.99	**-.15**	**	.01	.85
Childhood MHT	Older-age BMI	388	-.04	.44	-.01	.81	***	.94

*BMI* body mass index, *MHT* Moray House Test, *NART* National Adult Reading Test, *RSPM* Raven’s Standard Progressive Matrices. Statistics reported are standardised *β* coefficients and uncorrected p values derived from generalised linear models. All variables were residualised over age at measurement before modelling. Although variables are modelled as ‘predictor’ and ‘outcome’ variables, direction of causation is not implied, particularly for contemporaneous associations. Significant results at *p* < .05 are highlighted in bold; ** *p* < .01; *** *p* < .001

Again, as for height, we first looked at the relationship between BMI and intelligence at age 11 (Additional file 1: Figure S1, bottom). There was no significant relationship between childhood BMI and intelligence for any of the sub-samples. No two sub-samples significantly differed from one another in terms of the strength of this correlation (all *p* > .196). Among those members of the 36-Day Sample who completed cognitive testing in late adulthood, there was no significant correlation between older-age BMI and performance on either the NART or RSPM (Table 3).

We next assessed the longitudinal relationship between BMI and intelligence. Firstly, BMI, was less stable from childhood to late adulthood than height or intelligence, but still showed a moderate and significant association over time (*β* = .26, *p* < .001), which remained significant after correcting for multiple comparisons. The relations between childhood and late adulthood measures of intelligence were reported above and included in Table 2, confirming a strong positive correlation in each case. However, the negative relationships between childhood BMI and older-age NART performance, and between childhood MHT performance and older-age BMI were not strong enough to achieve significance, and neither did childhood BMI predict late-adulthood RSPM performance. There were significant effects of sex in models of performance on the MHT (*β* = .12, *p* = .02) and RSPM (older-age BMI: *β* = −.14, *p* < .001; childhood BMI: *β* = −.15, *p* < .001), but no significant interactions.

## Discussion

In this study, we assessed the relations between height and intelligence, and between BMI and intelligence, in childhood, in late adulthood and over the interval of up to 67 years in between. Sex was expected to influence longitudinal relationships, at least between height and intelligence, so was included as a covariate in all analyses. At age 11 years, there were modest positive associations between height and IQ in each sub-sample. There were similar associations between height and measures of two different domains of cognitive ability in late adulthood among those followed up. Height and intelligence both showed reasonable stability from childhood to older age. Childhood height also predicted older-age non-verbal reasoning and particularly verbal ability, and childhood intelligence also predicted older-age height. Sex showed a significant effect in many of these models, but there was no significant interaction between sex and the main predictor for any of the models – except that which assessed the association between childhood height and older-age height. Further testing confirmed that this association was, nonetheless, similarly strong in men and women assessed separately. Late-adulthood BMI correlated with childhood BMI, but we found no evidence of a relationship between intelligence and BMI in childhood, in late adulthood, or across the lifespan.

Our results support previous evidence of a contemporaneous relationship between height and intelligence. For example, Kanazawa and Rayniers [1] observed a significant (although small) correlation of around .11 between height and intelligence in a large sample of young adults. Beauchamp et al. [2] found the correlation to be slightly stronger at .18 in a sample of 28 to 55-year-old Swedish twins. However, at least during childhood, we observed slightly higher height-intelligence correlations of between .23 and .26 across sub-samples. These figures are comparable to those of Humphreys et al. [3], who also studied the relationship in children, and found correlations of between .25 and .35 at age 11 years. In a study of 160 children, also of a mean age of approximately 11 years, Taki et al. [5] found similar strength correlations between height and measures of IQ of around .26. Taki et al. suggest that the relationship between height and IQ is particularly strong in childhood due to parallel development of the two; more specifically, because increases in intelligence are linked to physical growth via brain development. Yet we still observed a stronger relationship between height and cognitive ability in late adulthood than others have measured in earlier adulthood: .29 for verbal ability and .21 for non-verbal reasoning. This may have a similar explanation, i.e. height and intelligence may be more closely related in late adulthood due to a link between declines in both measures. Indeed, Starr et al. [6] demonstrated that cognitive decline predicts greater loss in height in late adulthood. Starr et al. found a contemporaneous correlation of only .15 at age 79 years (similar to the age of the participants in the present study), but this would not be significantly different from our slightly higher results.

As height and intelligence relate to one another both in childhood and in late adulthood, possibly due to common mechanisms underlying their development and decline, one would expect there to be a longitudinal relationship between the two. Humphreys et al. [3] observed longitudinal cross-correlations between height and intelligence of .17 and .25, but this was only over nine years, in girls aged 8–17 years. Abbott et al. [33] made use of a much larger sample of Japanese men who had migrated to Hawaii, born in the first two decades of the 20th century. Over 8000 had their height recorded between 1965 and 1968, at a mean age of approximately 53 years. Around 25 years later, just under half of the original cohort were screened for poor cognitive performance. Those men who were shorter in middle-age were considerably more likely to perform poorly on the cognitive screening test – 25 % of those below 152 cm tall in middle-age fell below the criterion, compared to only 9 % of those above 173 cm – demonstrating a relationship between height and intelligence over 25 years. Similarly, Russ et al. [34] observed a dementia death hazard ratio of 1.24 per SD decrease in height. In this study, we assessed the relationship over a far greater interval of up to 67 years and observed a significant association of .20 between early height and later verbal ability, a slightly weaker association of .14 between childhood height and older-age non-verbal reasoning, as well as a significant correlation of .12 between childhood intelligence and late-adulthood height. Overall, our results are therefore consistent with previous findings, as well as our original hypotheses.

A longitudinal relationship between height and intelligence could simply reflect a combination of a contemporaneous height-intelligence relationship and the stability of one construct or the other. For instance, childhood height may predict older-age cognitive ability because height and cognitive ability are related in childhood, and cognitive ability is relatively stable across the lifespan. However, the correlations between childhood height and older-age cognitive ability were greater than the products of the correlations between childhood height and cognitive ability, and between childhood and older-age cognitive ability. It may be that the longitudinal relationship between height and intelligence is more complex, with each influencing the development of the other across the lifespan. For example, higher childhood intelligence may promote the adoption of a more nutritious diet throughout life, which could increase growth during adolescence, and perhaps also reduce loss of height in older age. Alternatively, or perhaps concurrently, being taller could give a child greater confidence, which could benefit their education and increase their social participation, facilitating intellectual development in early life and protecting against cognitive decline in later life. Declines in height and intelligence may also share some common mechanisms in older age. For example, physical exercise is thought to both attenuate height loss [35] and protect against cognitive decline [36] in older people. Similarly, elevated cortisol levels, most likely due to exposure to stress, have been shown to accelerate bone loss [37], largely responsible for ageing-related height decline, and are also thought to accelerate brain ageing and cognitive decline [38, 39]. Senescence-related genes could also promote declines in both height and intelligence. These accounts are highly speculative of course, but they provide examples of how height and intelligence could potentially influence each other across the lifespan. Further investigation is required in order to understand the true underlying mechanisms.

Some prior studies of the longitudinal relationship between height and intelligence have found that associations depended upon sex. For example, Humphreys et al. [3] observed a stronger longitudinal relationship between height and intelligence in girls than boys, while Quan et al. [7] observed the exact opposite in late adulthood: a stronger relationship in men than in women. We therefore investigated the role of sex in all of the associations assessed, but only observed a significant (before correcting for multiple comparisons) interaction with sex for the association between childhood and older-age height. We assessed this association in men and women separately, observing similarly strong associations in the two groups. Our results therefore do not suggest that the lifelong association between height and intelligence differs by sex.

Previous work has demonstrated a negative correlation between BMI and intelligence, particularly in late adulthood [12–14], although the direction of causation is unclear. Some reason that more intelligent people generally eat more healthily and exercise more regularly, thus maintaining a healthier BMI [17–19], while others suggest that obesity and associated vascular risk factors actually cause cognitive decline [14–16]. Our results provide no insight here, as we observed no significant association between childhood intelligence and late-adulthood BMI, nor between childhood BMI and late-adulthood cognitive ability. Further, we found no evidence in support of a contemporaneous association between BMI and intelligence, in childhood or in late adulthood. While this is consistent with some previous findings [5], we do not infer from our results that there is no relationship between BMI and intelligence. Instead, it is likely that the relationship is complicated by other factors, such as social class [40] and education [17], and as a result, in this case, was not detected.

The SMS1947 included almost every Scottish child born in 1936, and as members of the 36-Day Sample were selected according to their dates of birth being on 36 days throughout the year, this sample was incredibly representative of the population [23, 27]. However, participation in the follow-up study was dependent, firstly, upon participants still being alive and reachable in 2012–13, and, secondly, on their agreeing to take part in the study. As a result, approximately 12 % of the original sample took part in the follow-up study, with only around 6 % providing both physical and cognitive measures in late adulthood, and these sub-samples could not be expected to be anywhere near as representative of the population. Although we found no significant differences between sub-samples in terms of the strength of correlations between childhood IQ and physical metrics, the sub-samples did differ in important ways, for example, those who participated in late adulthood were significantly taller and heavier, and considerably more intelligent as children than those who did not. However, although the selectivity of our follow-up sample is a limitation of this study, the restriction in range that it introduced – especially as those who did participate in the follow-up were taller and more intelligent – most likely reduced the strengths of the assessed correlations. Had we been able to measure older-age height and intelligence in all of the original 36-Day Sample, we may have observed even stronger lifelong associations.

The selective pressures affecting the representativeness of the follow-up sample also led to the sample being relatively small. As above, we suspect that this may at least partly explain why we did not detect any relationship between BMI and intelligence. Additionally, our study was limited by the measurements of height and weight being taken by a number of different assessors in childhood, and by the participants themselves in late adulthood. However, in assessing a sample spread throughout the entire country of Scotland in childhood, and the even wider area of Great Britain in late adulthood, this was difficult to avoid. We also believe that, although measurement errors would not have been consistent across participants, they would not have been systematically related to intelligence.

## Conclusions

Both height and BMI have already been demonstrated to relate to intelligence contemporaneously and over periods of several years. Although we found no significant associations between BMI and intelligence, we observed that the relationship between height and intelligence is significant even over an interval of as much as 67 years. We also observed that, despite a large sex difference in late adulthood height, the lifelong associations between height and intelligence were not substantially influenced by sex. Further research is required to understand the mechanisms underlying the lifelong association between height and intelligence.

## Additional file

Additional file 1: Figure S1. Height, body mass index and intelligence at age 11 years by sub-sample. MHT IQ = Moray House Test intelligence quotient; BMI = body mass index. Correlations between height (top/blue) and BMI (bottom/red), and IQ at age 11 are represented for members of the 36-Day Sample who participated in the Scottish Mental Survey of 1947 (*N* = 5,742), for those who also provided physical measurements in older age (*N* = 392) and those who also completed cognitive testing (*N* = 343). (TIF 547 kb)

## Abbreviations

BMI: Body mass index
MHT: Moray House Test
NART: National Adult Reading Test
NHSCR: (United Kingdom) National Health Service Central Register
RSPM: Raven’s Standard Progressive Matrices
SMS1947: Scottish Mental Survey of 1947
