# Supplementary figures and images for: Associations among height, body mass index and intelligence from age 11 to age 78 years
Source: BMC Geriatr. 2016 Sep 29;16:167. doi: 10.1186/s12877-016-0340-0 (PMC5041406; doi:10.1186/s12877-016-0340-0)

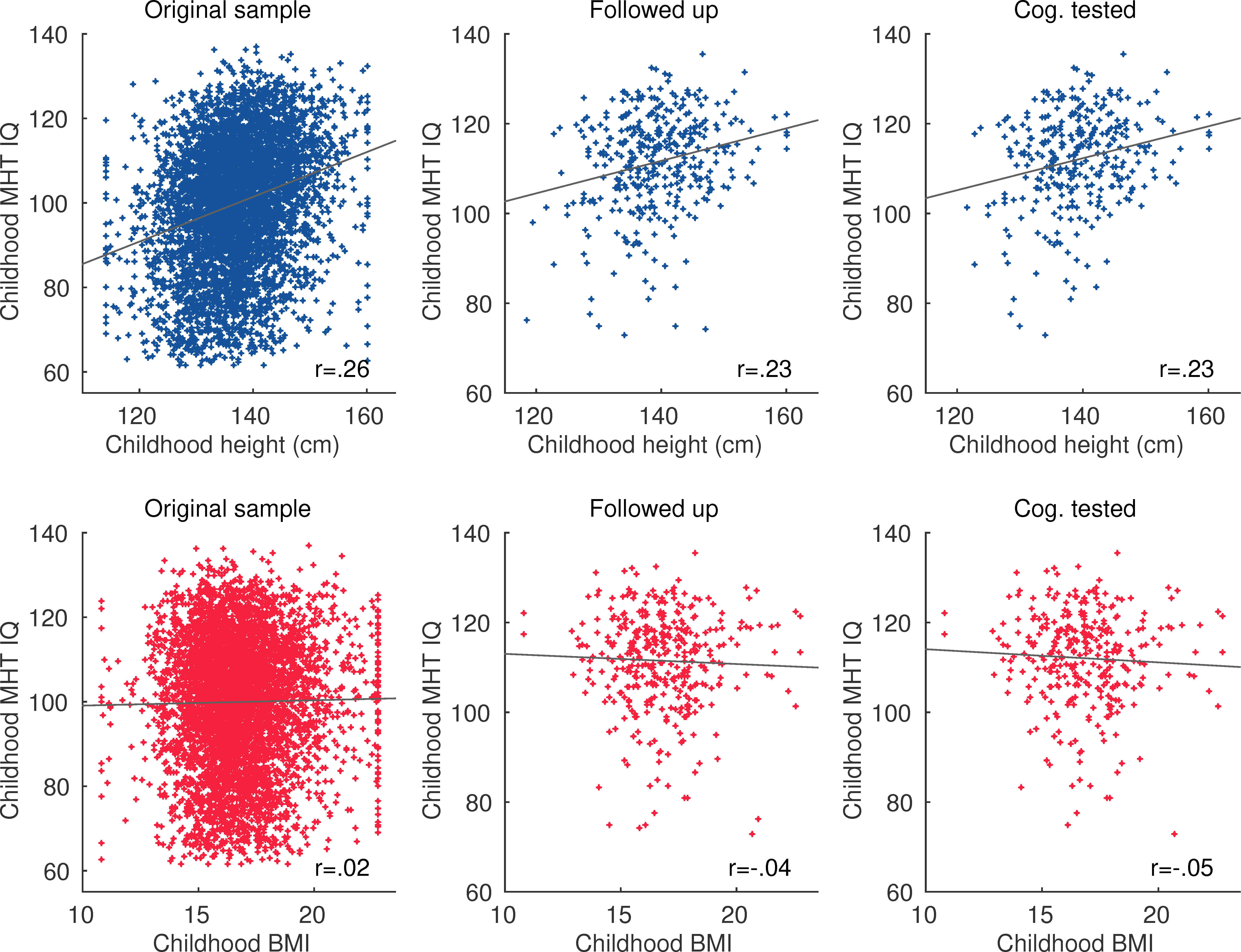

Supplement: Additional file 1: Figure S1. — Height, body mass index and intelligence at age 11 years by sub-sample. MHT IQ = Moray House Test intelligence quotient; BMI = body mass index. Correlations between height (top/blue) and BMI (bottom/red), and IQ at age 11 are represented for members of the 36-Day Sample who participated in the Scottish Mental Survey of 1947 (N = 5,742), for those who also provided physical measurements in older age (N = 392) and those who also completed cognitive testing (N = 343). (TIF 547 kb) [file 12877_2016_340_MOESM1_ESM.tif]
